# Osteoimmunology of Spondyloarthritis

**DOI:** 10.3390/ijms241914924

**Published:** 2023-10-05

**Authors:** Angelo Fassio, Fabiola Atzeni, Maurizio Rossini, Valeria D’Amico, Francesco Cantatore, Maria Sole Chimenti, Chiara Crotti, Bruno Frediani, Andrea Giusti, Giusy Peluso, Guido Rovera, Palma Scolieri, Vincenzo Raimondo, Davide Gatti, on behalf of the Study Group on Osteoporosis and Skeletal Metabolic Diseases of the Italian Society of Rheumatology

**Affiliations:** 1 Dipartimento di Medicina, Università di Verona, 37124 Verona, Italy; maurizio.rossini@univr.it (M.R.); davide.gatti@univr.it (D.G.); 2 Unità Operativa Complessa di Reumatologia Azienda Ospedaliero Universitaria Policlinico “G. Martino” di Messina, 35128 Messina, Italy; atzenifabiola@hotmail.com (F.A.); damico.valeria@libero.it (V.D.); 3 Unità Operativa Complessa di Reumatologia Universitaria, Polic. “Riuniti” di Foggia, 71122 Foggia, Italy; francescopaolo.cantatore@unifg.it; 4 Dipartimento di Medicina dei Sistemi, Reumatologia, Allergologia e Immunologia Clinica Università di Roma Tor Vergata, 00133 Rome, Italy; maria.sole.chimenti@uniroma2.it; 5 UOC Osteoporosi e Malattie Metaboliche dell’Osso Dipartimento di Reumatologia e Scienze Mediche ASST-G. Pini-CTO, 20122 Milan, Italy; doc.chiara.crotti@gmail.com; 6 Department of Medical, Surgical and Neuroscience Sciences, Rheumatology University of Siena, 53100 Siena, Italy; bruno.frediani@unisi.it; 7 SSD Malattie Reumatologiche e del Metabolismo Osseo, Dipartimento delle Specialità Mediche, ASL3, 16132 Genova, Italy; andreagiusti6613@gmail.com; 8 UOC di Reumatologia-Fondazione Policlinico Universitario Agostino Gemelli-IRCSS, 00168 Rome, Italy; giusy.peluso@policlinicogemelli.it; 9 Ospedale S. Andrea, Divisione Reumatologia, 13100 Vercelli, Italy; guido.rovera.gr@gmail.com; 10 Ambulatorio di Reumatologia Ospedale Nuovo Regina Margherita ASL ROMA1, 00153 Rome, Italy; palma.scolieri@gmail.com; 11 Casa di Cura Madonna dello Scoglio, 88836 Crotone, Italy; vincenzoraimondo@hotmail.it

**Keywords:** osteoimmunology, spondyloarthritis, pathophysiology

## Abstract

The mechanisms underlying the development of bone damage in the context of spondyloarthritis (SpA) are not completely understood. To date, a considerable amount of evidence indicates that several developmental pathways are crucially involved in osteoimmunology. The present review explores the biological mechanisms underlying the relationship between inflammatory dysregulation, structural progression, and osteoporosis in this diverse family of conditions. We summarize the current knowledge of bone biology and balance and the foundations of bone regulation, including bone morphogenetic protein, the Wnt pathway, and Hedgehog signaling, as well as the role of cytokines in the development of bone damage in SpA. Other areas surveyed include the pathobiology of bone damage and systemic bone loss (osteoporosis) in SpA and the effects of pharmacological treatment on focal bone damage. Lastly, we present data relative to a survey of bone metabolic assessment in SpA from Italian bone specialist rheumatology centers. The results confirm that most of the attention to bone health is given to postmenopausal subjects and that the aspect of metabolic bone health may still be underrepresented. In our opinion, it may be the time for a call to action to increase the interest in and focus on the diagnosis and management of SpA.

## 1. Background

Spondyloarthritis (SpA) is a family of chronic rheumatic diseases characterized by inflammatory arthritis that may involve peripheral and/or axial joints [1,2,3]. SpA is currently adopted as an umbrella term comprising different pathological conditions such as ankylosing spondylitis (AS) and psoriatic arthritis (PsA). Albeit different, these diseases show a significant phenotypic overlap that sometimes raises questions in terms of differential diagnosis [4].

Axial-SpA (ax-SpA) is characterized by spinal pain and new bone formation which, in a subset of patients, may gradually lead to ankylosis of the axial skeleton [1]. Locally, one of the shared hallmarks typical of this SpA is enthesitis, namely, the inflammation of the tendon insertion sites into bone (enthesis) [5]. Enthesitis is currently considered among the fundamental pathological moments in the development of SpA and a key target of musculoskeletal inflammation in diseases such as PsA and ax-SpA, leading to inflammation, bone destruction, and pathologic bone proliferation [5].

The mechanisms underlying the development of bone damage in the context of SpA are not completely understood. To complicate the matter, the physio-pathological models of SpA need to account not only for pathological bone loss, a phenomenon typical of other rheumatic disorders such as rheumatoid arthritis (RA) [6], but also for pathologic bone formation [1].

To date, a considerable amount of experimental evidence indicates that several developmental pathways are crucially involved in this process. Furthermore, it has been acknowledged in recent years that the contribution of the immune system and the bone tissue are deeply intertwined, as demonstrated by the research of Takanagi in several papers and many other authors [7], with the coining of the term “osteoimmunology”.

## 2. The Origin of Osteoimmunology

It has been more than 20 years since the term “osteoimmunology” was first adopted in a “news and views” article in the journal *Nature* [8]. Once thought to be a rigid and unchanging entity, the bone tissue metabolism has been progressively reconceptualized as a dynamic process involving the secretion and resorption of the bone matrix, while new osteoactive compounds were developed for bone diseases [9]. The discovery of the fundamental importance of the receptor activator of nuclear factor-kb (RANK) provided the evidence that osteoblastic cells are capable of inducing osteoclastogenesis [10], and the strict relationship between the osteoclast and osteoblast lineage in this very process was further emphasized by the role of the macrophage colony stimulating factor (M-CSF), the lack of which causes an osteopetrotic phenotype [11]. In fact, cells belonging to the osteoblast lineage (osteoblasts and osteocytes) express both RANKL and M-CSF to support osteoclastogenesis [12].

Meanwhile, a particular molecule on the surface of activated T cells—named tumor necrosis factor (TNF)-related activation-induced cytokine (TRANCE) [13], now more commonly known as RANK-ligand (RANKL)—was found to activate bone-resorbing cells. The discovery of a fundamental osteoclastogenic mediator expressed by an immune cell line, constantly working to protect us from pathogens, raised a question: how can the human organism protect itself from pathogens without suffering from extensive bone loss? The understanding of the anti-osteoclastogenic role of the RANKL decoy receptor osteoprotegerin (OPG) [14] and of interferon-Y (IFN-Y) partially answered the question [15]. These brief remarks are sufficient to suggest the deep link between the skeletal and immune system and the birth and subsequent exponential growth of osteoimmunology. Unsurprisingly, this research field developed especially from the research on arthritis-related bone destruction and was pursued and investigated mainly by rheumatologists and bone biologists [16]. Over time, the accumulated data led to a nuanced understanding of the pathogenesis of bone damage in conditions such as RA [6] and the demonstration of the effectiveness of denosumab, an anti-RANKL monoclonal antibody, in the prevention of bone erosions in RA, as shown in a recent phase III randomized clinical trial (RCT) [17].

On the other hand, the pathogenesis of bone damage in SpA is less clear. One of the key points in these conditions is the ambivalent type of damage involving the skeleton, featuring both bone loss and pathologic bone formation. The present review will address the current perspectives on these subjects and the more recent findings.

## 3. Overview of Bone Biology and Balance

Bone homeostasis is the result of the net balance between bone resorption by osteoclasts and bone formation by osteoblasts [18]. Osteoclasts differentiate from bone marrow-derived monocyte–macrophage lineage cells and resorb bone via decalcification and matrix degradation mediated by the secretion of hydrogen ions and matrix-degrading enzymes [18]. On the other hand, the bone-forming cells, the osteoblasts, produce bone matrix proteins, mediate mineralization, and derive from the bone marrow-derived stem cell, also called mesenchymal stem cell (MSC) [18]. The differentiation of the MSC towards the osteoblastic lineage is regulated by a multitude of cytokines which influence the expression of cell lineage-specific sets of transcription factors [18]. As expected, genetic mutations in the development of MSC are associated with pathologic phenotype in vivo [19], and it is interesting to speculate whether conditions such as AS are characterized by dysregulated MSC differentiation and function.

The fundamental moment in osteoblast differentiation, also representing the point of convergence of many signal transduction pathways, is the activation of the transcription factor runt-related transcription factor-2 (Runx2) [20]. Runx2 is the master switch for osteoblast differentiation and influences a multitude of transcriptional activators and repressors, ending up with controlling the expression of osteoblast-specific genes including type I collagen (Col1), alkaline phosphatase (ALP), osteopontin (OPN), osteonectin (ON), and osteocalcin (OC) [20].

Runx2 also regulates the expression of the transcription factor osterix [21] which, in turn, furthers the osteoblast differentiation and function and regulates the expression of various osteoblast markers including osteocalcin, Dickkopf-1 (Dkk-1) and collagen type I α 1 chain (Col1a1) [22,23].

When the bone forming process is complete, some osteoblasts become embedded in the bone matrix, mature into osteocytes, and contribute to orchestrate both osteoclastic bone resorption and osteoblastic bone formation in response to mechanical and hormonal cues [24]. What then, are the cornerstones on which the bone balance is based upon, and how does their dysregulation lead to the bone damage characteristic of SpA?

## 4. The Cornerstones of Bone Regulation from the SpA Perspective

### 4.1. Bone Morphogenetic Proteins

The bone morphogenetic proteins (BMPs) are growth factors and cytokines, originally identified as proteins able to induce the full cascade of endochondral bone formation [25]. Currently, BMPs are considered members of the superfamily of transforming growth factor beta (TGF-β) [25]. The best-known downstream signals to the primed BMP protein activation event are the small mother against decapentaplegic (Smad) proteins and the eventual activation of mitogen-activated protein kinases (MAPKs) such as p38 and extracellular signal-regulated kinases (ERK) [26]. BMP signals are mediated by type I and II BMP receptors and their downstream molecules, small mother against decapentaplegic (Smad). Phosphorylated Smad proteins form a complex that eventually is translocated into the nucleus, where it interacts with other transcription factors such as Runx2 [26]. More than 30 BMPs have been identified to date, but only a few of them have been investigated in the pathogenesis of SpA and seem to participate in different stages of the development of structural damage [26]. BMP-2, 6, and 7 are probably involved in different stages of the enthesitis process [27] and MSC differentiation into osteoblasts [28].

As one of the main regulators of BMP signaling, the secreted polypeptide noggin, encoded by the NOG gene, binds with BMP-2, 4, and 7 to block the BMP cascade [26].

In mice, imbalance between BMP-2 and noggin secretion induces abnormal osteogenic differentiation of AS-MSCs [29] and, in humans, autoantibodies binding noggin, therefore unleashing BMP signaling, which has been detected in healthy individuals but occurs at significantly higher levels in AS patients [30]. In a series of studies on BMP signaling, Lories et al. documented that its inhibition by noggin resulted in protection against arthritis and ankylosis in inflammatory models, not only in a preventive setting (before the onset of disease) but also in a therapeutic setup at the onset of clinical symptoms [31]. Subsequent ex vivo analysis using biopsy material from SpA patients with enthesitis further corroborated the observation that BMP signaling is active in the early stages of MSCs’ commitment [31].

In this regard, a meta-analysis published in 2019 retrieved five studies on serum BMP-2 in AS patients and confirmed the presence of increased levels of the molecule [32]. BMP-2 has been therefore proposed as a possible biomarker correlated with clinical and radiological indexes [33]. As promising as it might be, longitudinal data are needed to verify the predictive usefulness of BMP-2 as a biomarker for screening and/or monitoring patients at higher risk for structural progression.

### 4.2. The Wnt Pathway

**Figure 1:**
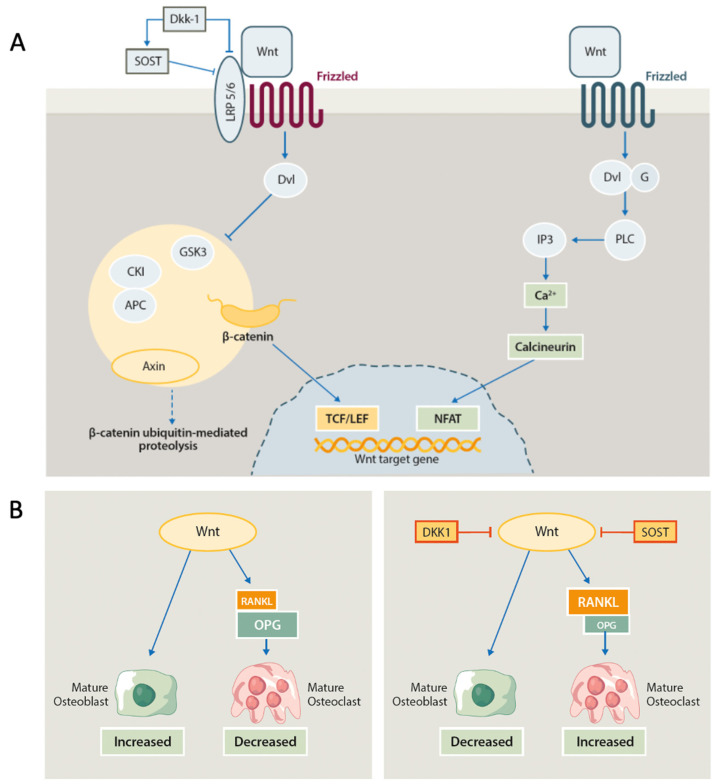
(**A**) Wnt canonical pathway: Wnt proteins bind to their co-receptor complex (Frizzled and low-density lipoprotein receptor-related protein 5/6) leading to inhibition of glycogen synthase kinase-3 (GSK3), mediated by dishevelled (Dvl) protein. Following this, β-catenin can translocate into the nucleus and, together with T cell factor (TCF)/lymphoid enhancer factor 1 (LEF), induces expression of Wnt target genes. Wnt non-canonical (Ca2+) pathway: Wnt proteins binding to the Frizzled receptor also leads to the activation of Dvl via activation of G proteins (G). Dvl leads to cytoplasmic calcium (Ca^2+^) release from the endoplasmic reticulum via phospholipase C (PLC) and inositol 1,4,5-trisphosphate (IP3). Intracellular Ca^2+^, in turn, activates calcineurin, which activates the nuclear factor of activated T cells (NFAT), inducing the expression of Wnt target genes, leading to osteoblast differentiation. (**B**) Activation of the Wnt canonical pathway leads to osteoblast differentiation, upregulation of osteoprotegerin (OPG), and downregulation of receptor activator of nuclear factor-kappa B ligand (RANKL). Dickkopf (Dkk) and sclerostin (SOST) inhibit the Wnt canonical pathway by binding LRP5/6 and preventing the formation of the co-receptor complex and subsequent Wnt canonical signal transduction. In this setting, β-catenin is degraded by a complex composed of GSK3, casein kinase I (CKI), adenomatous polyposis coli (PCI), and Axin.

The Wnt signaling pathway is essential for skeletal development and plays a critical role in homeostasis in adulthood [26]. This pathway has been implicated in osteoblastogenesis and is significantly affected by inflammatory responses. Since the seminal work of Diarra et al. in 2007 [34], it was clear that Wnt expression and inhibition by Dkk-1 and sclerostin was a critical knot in the development of bone damage in inflammatory conditions. In the presence of Wnt-specific ligands, β-catenin is protected by degradation, translocates to the nucleus, and ends up interacting with transcriptional activators, therefore influencing gene expression (Figure 1A) [26]. Dkk-1 and sclerostin bind to the Wnt co-receptor lipoprotein receptor-related protein (LPR) 5,6 and antagonize Wnt signaling [26]. The activated Wnt cascade promotes the progression of MSCs from osteoblast precursor cells into more mature osteoblasts while suppressing differentiation into adipogenic and chondrogenic lineages (Figure 1B) [35]. In the early phases following injury, β-catenin regulates the ratio of osteoblasts and chondrocytes present in the callus which arises from pluripotent MSCs [36]. In addition, the noncanonical β-catenin-independent Wnt pathway has also been shown to induce osteogenic differentiation through a different mechanism (Figure 1A). Non-canonical ligands such as Wnt5a have been shown to suppress the peroxisome proliferator-activated receptors-γ (PPARγ) [35]. Furthermore, Wnt5a seems to be the among the predominant Wnt expressed during osteoblastic differentiation of human MSC [37] and is increased in the early inflammation phase that takes place during fracture healing [38].

The interplay between the two distinct mechanisms induced by canonical and non-canonical Wnt ligands is still unclear, but as we’ve seen, it is now consolidated that Wnt, along with the other fundamental signaling pathways, regulates the osteogenic differentiation of MSCs. Dysregulation of the Wnt pathway is therefore one of the fundamental pathogenetic moments associated with bone damage in several conditions, as well as one of the most promising therapeutic targets [39].

There are a copious amount of data in mice models showing the effects of Wnt dysregulation in terms of bone and joint damage. The already mentioned paper from Diarra et al. demonstrated that Dkk-1 blockade with a monoclonal antibody prevented the development of erosions (but not inflammation) but was associated with the formation of osteophytes [34]. Similarly, in a mouse model of transgenic mice overexpressing TNFα, Dkk-1 blockade prevented erosions at the SIJ but enhanced the ankylosing process up to the joint fusion [40]. Apparently, Dkk-1 also seems to be downregulated in hip synovial tissues from AS patients compared to that observed in controls, with a consequent overexpression of Runx2, alkaline phosphatases ALP, and OC [41].

While in RA, a condition strictly characterized by bone loss, Wnt inhibition has been solidly replicated [42], the same finding is not as clear in SpA. In recent years, we investigated Wnt dysregulation in SpA, in particular in AS [43,44] and PsA [45], and documented decreased levels of serum Dkk-1 in both conditions, with an intriguing correlation between Dkk-1 and parathyroid hormone (PTH).

However, the overall data from the literature are still conflicting. In a meta-analysis published in 2018 including studies analyzing Dkk-1 serum levels vs. healthy controls, no significant difference was found [46]. Interestingly, in the subanalysis including studies involving patients with higher scores of structural damage, as measured by the modified stoke ankylosing spondylitis spinal score (mSASSS), Dkk-1 resulted in significantly reduced scores. In addition, there is evidence suggesting that Dkk-1 serum levels might be related to disease duration in SpA and, arguably, with increased structural damage [47].

A similar case could also be made for sclerostin, another fundamental inhibitor of the Wnt canonical pathway, expressed mainly (but not exclusively) by osteocytes [48]. Decreased expression of sclerostin was already demonstrated in 2009 by Appel et al., in a very elegant study in which they showed that sclerostin expression was virtually absent in the lesional osteocytes of subjects with AS [49]. Interestingly, low serum sclerostin levels in patients with AS were significantly associated with structural progression.

Conversely, a meta-analysis published in 2019 and including 18 studies did not show reduced levels of serum sclerostin in AS patients [32]. And yet, in the subanalysis dividing the studies according to continent of origin, sclerostin was indeed reduced in the European (N = 8) and South American (N = 1) [32] subgroups.

Clearly, different populations have different physical qualities and may vary greatly in terms of genetic and environmental characteristics. For instance, decreased levels of Wnt inhibitors (Dkk-1 and sclerostin) have been documented by Aschermann et al., not only in patients with SpA, but also in healthy subjects with HLA-B27 [50].

To interpret this variability, it is helpful to remember that both Dkk-1 and sclerostin have been shown to increase their expression under the effect of TNFα [34,51] and to decrease their expression after treatment with anti-TNFα monoclonal antibodies (TNFis) or glucocorticoids in different inflammatory rheumatic conditions [52,53,53] including AS [54]. In addition, the fluctuating uncontrolled inflammation (at each involved site) typical of SpA [55] and the influence of certain osteo-active hormones such as PTH [56] (known to be heavily influenced by vitamin D status [56]) should also be added into the equation, making it even more difficult to interpret a single observation of these biomarkers; this will be further discussed in a later chapter.

In our opinion, more than in diagnostic terms, these molecules should be investigated in longitudinal cohorts as prognostic markers, as already seen in the German spondyloarthritis inception cohort (GESPIC) [57], and, possibly, after careful evaluation of repeated measures over time.

### 4.3. Hedgehog Signaling

Hedgehogs (Hhs), of which there are three in mammals (i.e., sonic, Indian, and desert hedgehog), represent another fundamental node for bone development [58]. The cellular cascade to the Hh signals is initiated by two transmembrane proteins, the 12-transmembrane-spanning protein Patched-1 (Ptch) and the 7-transmembrane spanning receptor Smoothened (Smo). Smo has homology to G protein-coupled receptors and transduces the Hh signal [58]. In the absence of Hh, Ptch maintains Smo in an inactive state. With the binding of Hh, Ptch inhibition of Smo is released, and intracellular signaling is started [58].

In general, osteophytes (and, by extrapolation, enthesophytes) are formed through endochondral ossification. During this process, MSCs condense and enter the phase of chondrogenic differentiation, following a standard differentiation program (round, flat, and, eventually, hypertrophic chondrocytes) [59]. At the end, the hypertrophic cartilage is replaced by bone [59]. Ihh controls both the differentiation and proliferation of chondrocytes [60], and it is described as the master regulator of this process. Later on, Hh signaling regulates osteoblast differentiation by increasing the expression and function of Runx2 [61].

In addition, Hh signaling acts epistatically on both canonical Wnt signaling and BMP signaling in the osteoblast differentiation. Skeletal progenitors are specified into the Runx2-positive osteoblast precursor upon Hh input [60]. Canonical Wnt signaling then sequentially transitions the Runx2-positive osteoblast precursor into the osterix one [60]. Meanwhile, BMP signaling accelerates these processes after the Hh-mediated specification into the osteoblast lineage [60].

Hh signaling has not been extensively investigated yet in SpA, but experimental data on mice suggested that its blockade inhibits enthesophyte formation in an inflammatory model without affecting inflammation [62]. In another in vivo study, adopting an AS-like mouse model (CD4-Cre;Ptpn11^f/f^) characterized by kyphosis, scoliosis, arthritis, and bony fusion of axial joints, the authors observed that targeting dysfunctional chondrogenesis with a Smo inhibitor significantly reduced the AS-like bone disease in mice [63].

In 2015, Daoussis et al. first investigated this pathway in AS and observed that the serum level of Indian Hh (Ihh) in AS patients was higher than in RA patients and healthy controls, and its expression decreased after TNF-antagonist treatment [59]. Similar results were found for Dkk-1 and sclerostin. Aschermann et al. found higher levels of serum Ihh in healthy HLA-B27 individuals and SpA patients than HLA-B27-healthy controls [50].

As already mentioned, specific data on Hh signaling in SpA are still very scarce; on the other hand, this pathway has been recently suggested as a promising target to enhance bone healing [64]. In our opinion, given the relevance of Hh signaling on chondrogenesis and osteogenesis, its interaction with the Wnt and BMP pathway and its possible overexpression due to nuclear factor-kb activation [65] might support the importance of this pathway in the immunopathogenesis of bone damage in SpA and the importance of future studies on the subject.

## 5. Cytokines, the Immunologic Milieu, and Its Interplay in the Development of Bone Damage in SpA

Proinflammatory cytokines’ blockade by monoclonal antibodies currently represents the cornerstone of the treatment of SpA [66,67]. There is a significant body of studies in the literature showing how the immune cells, cytokines (i.e., TNF, IL-23, IL-22, IL-17 family members), and others are involved in tissue repair responses [68]. In addition, it is important to understand that inflammation is a necessary process that precedes bone formation and remodeling [69]. However, as often happens, when this physiological response is hijacked, pathology may ensue.

Fractured bone is repaired through a logical and sequential process characterized by inflammation, callus formation, and bone remodeling [69]. During the first moments of the repairment sequence, right after the formation of the hematoma, immune cells, including neutrophils, macrophages, and lymphocytes, infiltrate the injured site, and several proinflammatory mediators derived from the damaged tissues enhance the activity of these immune cells which, in turn, release proinflammatory cytokines at the injury site to propagate acute inflammation [69]. The hematoma works as a scaffold for recruited inflammatory cells and their secreted mediators including interleukin (IL)-1, IL-6, TNF-α, IL-17, C-C motif chemokine 2 (CCL2), M-CSF, and others [70]. Apoptotic lesional osteocytes release large amounts of RANKL [71] that, in concert with TNF-α [72], effectively stimulate osteoclast activity and the removal of the tissue debris. However, a proinflammatory cytokine such as IL-17 also serves another fundamental purpose: it enhances the MSCs’ proliferation and commitment to the osteoblast lineage [69].

Although beyond the scope of this review, it is important to mention the significance of genetic factors due to their essential role in this context. The influence of genetic susceptibility is not merely restricted to predisposition for dysregulated immune responses [73] but also extends to the development of bone maladaptive responses. For example, polymorphisms in BMPs have been linked to an increased susceptibility to AS and radiographic progression [74].

It is well known that HLA-B27 plays a pivotal role in the pathogenesis of AS, accounting for approximately 20% of AS heritability [75]. Furthermore, studies have shown an association between the presence of HLA-B27 and lower levels of Dkk-1 and sclerostin, even in healthy individuals carrying this allele [50].

Moreover, the biochemical and biophysical properties of HLA-B27 affect its propensity to misfold, leading to an endoplasmic reticulum stress response. This eventually triggers the so-called unfolded protein response (UPR) in cells [75]. Free heavy chains of HLA-B27 can induce inflammation via T cells, NK cells, and myeloid cells. The induction of UPR genes leads to the release of cytokines such as TNFα, IL-17, IL-23, and interferon-γ, along with an upregulation of Th17 cells [75]. Notably, in animal models of AS, HLA-B27 misfolding has been observed to activate the HLA-B27-mediated sXBP1/RARB/TNAP axis in AS MSCs within the enthesis. This axis plays a role in spinal ankylosis and contributes to the pathogenesis of syndesmophyte formation [76]. Intriguingly, the pathway leading to accelerated mineralization in AS MSCs is independent of Runx2, suggesting that in this model, syndesmophyte formation might not solely rely on standard osteoblast-mediated bone remodeling.

In addition to HLA-B27, recent genome-wide association studies (GWASs) over the past decades have identified several other polymorphisms associated with increased risk for bone damage and radiographic progression in SpA [77]. These discoveries underscore the notion that in predisposed individuals, dysregulation of bone metabolism is not solely attributed to immune dysregulation and the aberrant production of cytokines and bone-active mediators. It may also stem from an inherent tendency of MSCs and osteoblasts to exhibit abnormal responses under certain conditions.

## 6. Cytokines Involved in Bone Damage and Tissue Repair

### 6.1. The Central Role of IL-17

As is known, aberrantly regulated T helper (Th) 17 immunity is a critical component in inducing AS phenotype [78,79], with increased levels in the number of Th17 cells in the peripheral blood and synovium of AS patients [78,80].

The polarization towards Th17 cells from naïve CD4 T cells is operated by several cytokines, including IL-1β, IL-6, transforming growth factor-beta (TGF-β), and IL-23 [78]. Aside from producing IL-17, Th17 have been observed to produce other mediators involved in pathologic bone formation, such as IL-22 [78].

IL-22 has been shown to enhance the osteogenic capacity of MSC in vitro in an inflammatory environment [81] while, in the same model, the presence of IFN-γ and TNF suppressed osteogenesis. Interestingly, mouse osteoblasts express low levels of the IL-22 receptor α, but its expression seems to be upregulated when primed with BMP-2 [68]. However, while the role of IL-22 seems intriguing, the evidence supporting its role in SpA is still limited.

The detection of spinal entheseal γδ T cells in humans that produce IL-17 independent of IL-23 [82] may help to explain the discrepancy between recent clinical trials and preclinical studies (i.e., the negative results of the anti-IL-12/23 ustekinumab in a phase III trial in axial-SpA [83].

One second source of IL-23 independent IL-17 production has been suggested in Th17 cells after stimulation with prostaglandin E2 (PGE2) [84,85]. Once again, this link seems relevant if we consider that nonsteroidal anti-inflammatory drugs (NSAIDs) and cyclooxygenase-2 inhibitors (COXIBs) are still a recommended treatment in both axial and peripheral SpA [66,67], albeit with inconsistent results in terms of inhibition of radiographic progression (more on this subject in a following paragraph). Furthermore, PGE2 has been shown to positively interact with BMP [86], Wnt [87], and Hh signaling [88] in different models.

The role of other metabolic pathways in the regulation of the proinflammatory cytokine pathways cannot be excluded as well. Recently, exploratory data on an open-label randomized trial with cholecalciferol in a population of healthy vitamin D-deficient subjects showed a decrease in IL-17A and IL-6 after cholecalciferol supplementation [89]. Interestingly, the Vitamin D and Omega-3 Trial (VITAL), a nationwide, randomized, double-blind, placebo-controlled trial on vitamin D and omega-3 fatty acids, recently reported that cholecalciferol supplementation (daily 2000 IU) for five years, with or without omega-3 fatty acids, reduced the incidence of new autoimmune disease by 22% in a low-risk healthy population [90]. In addition, the biochemical data from the same trial showed, in multiple-adjusted models, that cholecalciferol supplementation decreased serum high sensitivity C-reactive protein levels by 19% at two-year follow-up, albeit without major differences in IL-6, IL-17, and TNFα (IL-17 was not investigated) [91].

How does IL-17 promote osteogenesis? IL-17A, in particular, that produced by γδ T cells, is able to exert both positive and negative effects on osteoblastogenesis, depending on the target cell types and on the advancement of their differentiation program’s positive effects on immature mesenchymal cells including MSCs, myoblasts, and mesenchymal cells at the bone injury site. On the other hand, negative effects on calvarial pre-osteoblasts [69].

IL-17A and IL-17F have been shown to stimulate the osteogenic differentiation of progenitor cells via Runx2 and osterix expression in vitro, both through synergy with BMP-2 (77,78) and through BMP-independent [92] signaling.

In addition, IL-17 seems to exert its influence on the Wnt pathway. In MSC isolated from the bone marrow of AS patients, Dkk-1 was observed to be underexpressed and further inhibited by IL-17 [93]. A similar inhibition of Dkk-1 expression was also observed in other models [94,95,96]. In humans, inhibition of IL-17 with the administration of secukinumab in patients with peripheral PsA has been associated with a normalization of Dkk-1 serum concentration and an increase in sclerostin [97]. Recently, data on serum sclerostin concentrations after treatment with secukinumab (from the MEASURE-1) have been published [98]; no significant changes were found at week 52 and 104. However, such late observations are difficult to interpret, as the kinetics of Wnt regulations might reveal changes in a much quicker fashion. For instance, changes in Dkk-1 have been shown as early as seven days after the first administration of a TNFi in RA [52]. On the other hand, treatment with the anti-sclerostin monoclonal antibody romosozumab in postmenopausal osteoporosis showed dramatic acute response in terms of bone turnover markers (BTMs) that, however, quickly plateaued and returned to baseline at the 12th month [99]. Further studies are needed to investigate the effects of IL-17 blockade on the Wnt system in SpA patients and to verify if their presence is associated with improved structural outcomes.

IL-17 is also involved in osteoclastogenesis and consequent bone loss. Th17 cells accumulate in the synovium and produce IL-17A/IL-17F, which stimulates RANKL expression on synovial fibroblasts and, at the same time, activates synovial macrophages so that they secrete inflammatory cytokines such as TNFα and IL-6 [16].

In conclusion, considering that IL-17A is highly upregulated in the period immediately after fracture, it seems likely that this cytokine positively regulates the early phase of osteoblastogenesis [69] and, at the same time, stimulates bone resorption and amplifies inflammation [16], therefore contributing to the removal of debris while paving the way for the subsequent repair process. These considerations may help in understanding apparently inconsistent results when investigating the overall effects of IL-17. Defined as a “fine tuning” cytokine, its effects appear to be context- and timing-dependent [69,100].

### 6.2. Interleukin-6

IL-6 is a renowned cytokine involved in a range of biological processes [68]. The significance of IL-6 in the pathogenesis of AS is questioned by the lack of efficacy in AS of monoclonal antibodies binding the IL-6 receptor [101]. However, IL-6 seems to play a role alongside the IL-23/17 axis, where it seems to be considered a key driver in the differentiation of de novo Th17 T cells in conjunction with IL-23 [68].

Serum IL-6 concentrations have been shown to be increased in AS subjects [102,103] and to decrease after treatment with TNFi [103]. After a fracture event, IL-6 is expressed by osteoblasts; then, its levels tend to decrease over the course of the fracture repair [69].

IL-6 signaling occurs via the Janus kinases (JAK)/signal transducer of activation (STAT) signaling pathway [68]. In particular, the activation of STAT3 in fibroblasts and osteoblasts promotes the expression of RANKL, fosters osteoclasts’ differentiation and activation, and enhances inflammation and joint destruction [104]. At the same time, the activation of STAT3 seems to stimulate the activation of Th17 cells in AS patients with peripheral arthritis [105].

As already mentioned, however, the clinical significance of IL-6 in SpA is still poorly defined, and although its role might not be a central one in these conditions, it is likely that it contributes to osteoclast activation and local and systemic bone loss.

### 6.3. TNFα

Certainly, another fundamental cytokine in the pathogenesis of SpA is TNFα. TNFα is mainly produced by activated macrophages and binds to TNF receptor 1 (TNFR1) and TNFR2 [69]. TNFR1 is ubiquitously expressed, and its signal induces the activation of caspase-8, NF-kB, and MAPKs, leading to apoptosis and inflammation, while TNFR2 is highly expressed by immune cells, and its signaling activates NF-κB and the MAPKs [69].

Importantly, the Wnt pathway cross-talks with inflammatory signaling processes during bone formation. TNFα has been shown to induce Dkk-1, thus blocking osteoblast differentiation [34,51]. The profound inhibition of the Wnt canonical pathway is also associated with a shift in the RANKL to OPG ratio and, therefore, not only with an increase in bone resorption, but also with impaired bone repair [39]. On the other hand, neutralizing Dkk-1 with monoclonal antibodies in TNFα transgenic mice inhibited the joint destruction and resulted in osteophyte formation [34]. In other words, the balance between bone formation and bone resorption is strongly influenced by the cross-talk between the Wnt signaling and the TNFα-induced inflammatory process.

Interestingly, however, in various in vitro experiments in which MSCs were treated with TNFα, TNFα also showed either a promoting or suppressing effect on bone formation [69], which, once again, might be explained by differential expression patterns of TNFα receptors, the different local concentration of this cytokine [106], and the state of the other signaling molecules. While the effects on the osteoblast lineage are still controversial, it is clear that TNFα plays a role in osteoclast development, ultimately by increasing RANKL expression and by directly stimulating and inducing osteoclast differentiation in the presence of RANKL [16].

As already mentioned, TNFα blockade has been associated with decreases of Dkk-1 (and sclerostin) in different inflammatory rheumatic conditions [52,53,54,107,108]. In a study published in 2019, Zhao et al. observed a reduction of Dkk-1 in SpA patients after treatment with TNFi, and these changes were also significantly correlated with the baseline and changes in spinal bone marrow edema (BME) levels in magnetic resonance imaging (MRI) [109], an imaging hallmark of active inflammation in this condition. TNFα expression has been proved to be significantly increased in areas of BME of ax-SpA patients, as proved by human data investigating the ^99^m-Technetium (Tc^99^m)-labelled TNFi antibody uptake in BME areas [110]. However, the overall data on TNFi treatment and Wnt inhibition in SpA are somewhat conflicting, with studies showing a decrease in Dkk-1 [54,109], an increase [111], or stable levels [112,113].

This heterogeneity might be explained by the different assay performances by various manufacturers, lack of standardization, and possible analytical issues that can affect the accuracy of immunoassays [114,115], but also by the site-specific fluctuating bouts of inflammation/repair that typically characterize SpA [55] and, therefore, influence the net balance of a serum biomarker. This topic will be addressed in the next paragraph.

**Figure 2:**
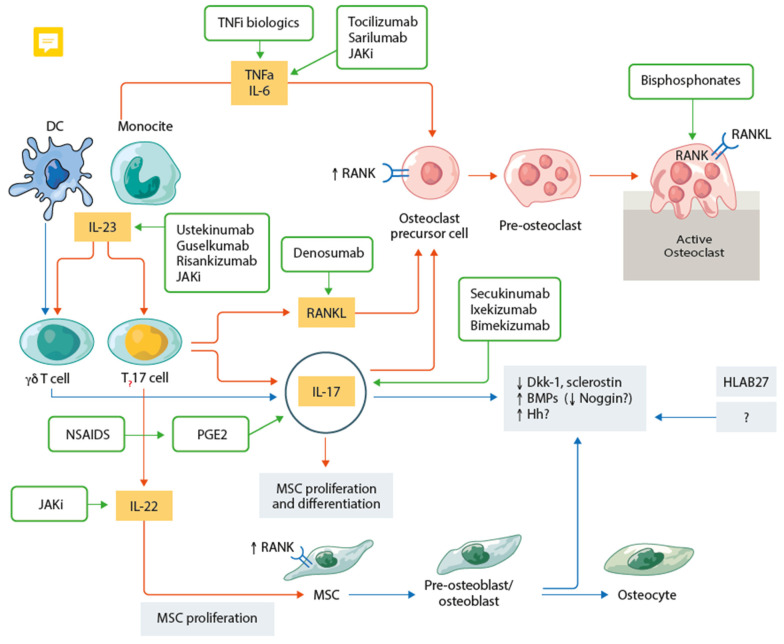
The cellular physiopathology of bone damage in spondyloarthritis. Different cytokines act in concert in the development of lytic and productive bone damage; in particular, dysregulated IL17 expression may act both by stimulating the osteoclast activity and by priming the multipotent bone marrow-derived stem cell (also called mesenchymal stem cell) towards the osteoblast lineage. The balance of several bone regulators (i.e., Wnt inhibitors, Noggin, BMPs, etc.) determines the final local and systemic net effects. The figure also includes the targets of several currently available drugs for the management of inflammatory arthritides.

The cytokine network leading to bone biology dysregulation and damage is summarized in Table 1 and Figure 2.

## 7. The Pathobiology of Bone Damage in SpA

**Figure 3:**
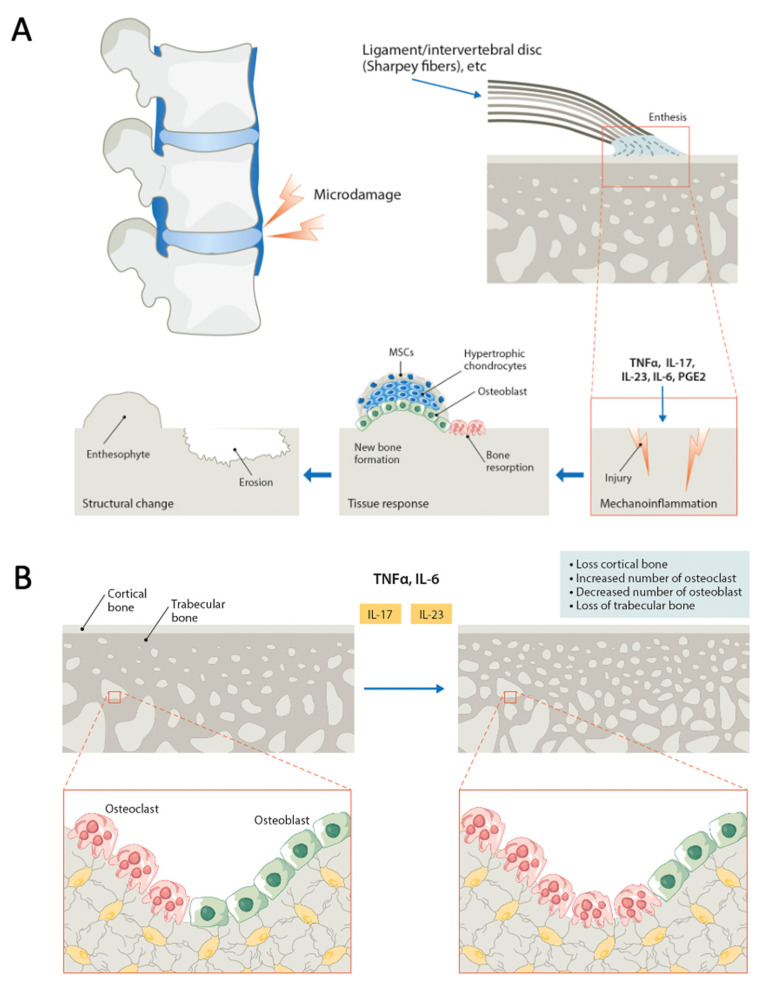
(**A**) In patients with clinical or preclinical SpA, mechanical strain at enthesial sites may be followed by mechano-inflammation and subsequent development of enthesitis, which is induced and amplified by proinflammatory mediators. Afterwards, the dysregulated environment may evolve with the development of both pathologic bone formation (syndesmophytes, enthesiophytes), and athologic (local) bone resorption (erosions). (**B**) Chronic systemic inflammation is associated with impaired bone health due to the overexpression of proinflammatory cytokines and RANKL. These mediators, over time, cause a systemic increase in bone turnover, with a negative balance. The net result is osteoporosis, with reduced cortical and trabecular thickness, increased cortical porosity, and reduced trabecular connectivity.

As already mentioned, bone health (or damage) is the net result of bone resorption vs. bone formation. In the setting of bone fracture healing, inflammation is followed by an orderly sequence of events that culminate with bone repair and is essential for the priming of MSCs toward the osteoblast lineage. Pathology hijacks these mechanisms, and in SpA uncontrolled inflammation, unsurprisingly associated with bone loss (i.e., erosions), is followed by pathologic ossification at the entheseal sites [55], eventually leading to the development of syndesmophytes, enthesophytes, and spine and joint ankylosis (Figure 3A). Simultaneously, a state of chronic inflammation is also associated with systemic deterioration of skeletal health (osteoporosis—see subsequent paragraphs and Figure 3B).

Though not always clearly documented, imaging, and in particular MRI, theoretically follows this sequence of events. Early lesions are characterized by subchondral BME in MRI [55] and erosions in X-rays [116]. More specifically, in 2009, Lambert et al. distinguished, in the spines of patients with AS, corner inflammatory lesions (CILs) type A, more recent and characterized by homogeneous short-tau inversion recovery (STIR)/fat-saturated T2 (T2FS) hyperintensity of the whole corner, and type B CIL, with a dimorphic appearance, characterized instead by hypointensity right at their edges [117]. Already, at that time, the authors hypothesized that a more advanced lesion (CIL B) could be associated with bone marrow reparative changes.

The next lesion, theoretically occurring when macroscopic inflammation expressed by BME is resolving or already resolved, is the fatty lesion, as documented by T1 hyperintensity in MRI [55,118]. Eventually, the overall process may lead to the appearance of a syndesmophyte and, therefore, structural progression. Several reasons make the case for the relevance of this sequence. First of all, while some studies somewhat showed an association between BME and structural progression [55], Maksymowych et al. suggested that the risk might be increased limitedly to the more advanced inflammatory lesions (CIL B) [117], especially under treatment with TNFi biologics, and, in another paper published in 2014, the same author observed that the resolution of inflammation and its reduction at the SIJ were each independently associated with the development of new fat metaplasia at two years [118].

Furthermore, in a cohort of 73 patients treated with TNFi, Baraliakos et al. documented that the corners more at risk for structural progression in the following five years of observation were those with fatty lesions already present at baseline (or that developed within the first two years) [119], and Machado et al. confirmed that both inflammatory (BME) lesions and fatty lesions were associated with radiographic progression, with a further increase in the combination of fat and inflammation either at the same time or in a sequential fashion [120].

Assuming that, in general, the more advanced the MRI lesion is, the higher the risk for structural progression, is there a histologic equivalent of these imaging findings? Sacroiliac joint (SIJ) biopsies in AS patients with BME lesions in MRI confirmed the presence of inflammatory changes with subchondral BME, with mononuclear cells commonly seen in proliferating connective tissue infiltrates [121]. Similar results were found in the spine in intervertebral joints [122], with interstitial CD3+ (either CD4+ or CD8+) T cells significantly more frequent in AS patients compared with non-AS control [123]. In addition, inflammatory bone marrow areas at histology, that may still be present within areas appearing as fatty lesions in MRI, have been shown to host significant numbers of active osteoclasts and a reduced (yet still somewhat significant) number of osteoblasts [124].

Interestingly, in biopsies of patients with AS, patients with early disease have been shown to have more T cells and to locally express higher levels of TNFα and IL-6, while patients with advanced disease showed more TGFβ [124]. In line with this remark, the inflammatory MRI lesions may subsequently evolve to fatty lesions, which are also characterized by extensive areas of granulation tissue with bony spots and significant cellular expression of Runx2 and Col1a1 [125]. In addition, within these areas, the authors also observed a reduced expression of sclerostin and Wnt inhibitor factor-1 (Wif-1), therefore suggesting the hyperactivation of the Wnt pathway [125]. A severe underexpression of Wnt inhibitors has been demonstrated in mouse models of AS, especially after the inflammatory phase [126].

Indeed, in the already mentioned study of Baraliakos et al., within MRI fatty lesions, and specifically in those spots histologically characterized by fatty bone marrow, a significant number of osteoblasts were found, along with a complete absence of osteoclasts [124].

On these premises, one current model to explain the development of bone damage in subjects developing SpA may first include an inflammatory phase, especially at the entheseal sites [5]. While acute/subacute inflammation thrives, osteoclast activity is enhanced by several actors (TNFα, IL-17, IL-6, RANKL). At the same time, key players such as PGE2 and cytokines (IL-17, IL-22, and others) may also trigger MSCs towards the osteoblastic lineage. Once the local inflammatory process has subsided (completely or partially) and the repairment phase is initiated, increased Wnt, BMP-2, TGFβ, and Hh signaling, under the influence of the above-mentioned molecules, in a predisposed setting (i.e., HLA-B27, impaired Wnt inhibition, etc.), might unleash a maladaptive bone production and, ultimately, lead to structural progression.

Nevertheless, it is necessary to understand that this sequential model inevitably oversimplifies what happens at the involved site; in our opinion, this process needs to be considered a continuum of phenomena that may, at least for a certain period of time, overlap and progressively fade from one phase to the next one at each region of interest (i.e., an involved vertebral corner). As usual, the net balance between the resorption and formation activity is what ultimately determines the destructive (i.e., erosions) or productive (enthesophytes, syndesmophytes) phenotype of bone damage.

Indeed, while a certain pathologic moment may prevail at the imaging examination (i.e., BME vs. fatty lesion), coexistence of inflammatory infiltrates and granulation tissue has been observed during the inflammatory phase [121], and, within fatty lesions, neighboring areas of osteoblast-enriched fatty bone marrow and of osteoclast-enriched inflammatory bone marrow have been described [124]. Furthermore, while nowadays not currently routinely recommended [127], bone scans (scintigraphy) with Tc99m-methylene bisphosphonate (Tc99m-MDP) have been used in the diagnosis of SpA [128]. It is acknowledged that bisphosphonates accumulate with high affinity in areas of the bone characterized by high turnover [129] and tend to bind well to resorption sites, presumably because calcium phosphate minerals are exposed during resorption [129]. The finding of positive bone scans in SpA subjects, therefore, suggests that the involved areas are characterized by increased bone turnover (with both resorption and osteoblastic activity), while, to date, there is no evidence of a complete local uncoupling of the two processes.

Similarly, [^18^F]fluoride positron emission tomography/computed tomography (PET/CT) has also been studied in the context of ax-SpA and has been shown to correlate with disease activity [130]. The main studies investigating [^18^F]fluoride PET/CT are summarized in Supplementary Table S1.

Unlike Tc^99^m-MDP, the [^18^F]fluoride isotope is a bone-specific tracer that, during bone remodeling, is incorporated into the skeleton at sites of active osteoblastic bone synthesis and, therefore, strictly highlights bone synthetic activity [130]. [^18^F]Fluoride uptake in anterior vertebral corners has been detected two years before the development of syndesmophytes in conventional radiographs [131]. If a strict dichotomous relationship between bone resorption/BME vs. bone formation/fatty lesion was expected, then [^18^F]fluoride uptake would be expected only in the T1-hyperintense fatty lesions. On the contrary, BME has also been associated with the presence of osteoblastic activity [132]. Furthermore, the combination of BME and FD showed the highest [18F]fluoride uptake, in line with a previously mentioned longitudinal imaging study that found the highest relative risk for progression in the BME plus fatty lesion corners [119,120]. Moreover, in a study published in 2015, Lee et al. documented CIL B to be the type of inflammatory lesions with the highest OR for [18] fluoride uptake (OR 95% CI: 59.9, 23.5–151.5, *p* < 0.001) [133].

Overall, these studies show that BME lesions are consistently associated with [^18^F]fluoride uptake, which corroborates the presence of an early triggering of osteoblastic activity within inflammatory lesions. Fatty lesions have been seldom investigated, and the small sample sizes and the lack of validated scores specifically addressing fatty lesions are currently limiting the extent of the conclusions that we can draw from the literature.

Finally, it is intriguing to speculate on the association between the accumulation of fat at critical sites (i.e., the spinal entheses) and pathological bone proliferation. This finding is puzzling, as osteoblasts and adipocytes both derive from the same MSCs, but their differentiation follows opposite pathways [134], and several studies have described an inverse relationship between adipogenesis and osteogenesis during MSC differentiation [135,136].

Firstly, we should consider that even MRI might fail to document prestructural lesions, as roughly up to 50% of syndesmophytes are apparently not preceded by MRI lesions. At least in part, this might be explained by the fact that the granulation tissue discussed earlier does not always show adipocytes and lipid accumulation at histology [125]. Clearly, in the absence of fat accumulation, no T1 hyperintense signal is likely to be detected in MRI. The mechanisms under which lipid accumulation may or may not happen are presently unclear.

In our opinion, it is currently difficult to hypothesize a causal link between the osteogenic maladaptive response and fat accumulation; further, in a study by Bleil et al., the extent of fat tissue was not associated with the presence of bony spots, while, conversely, this was true for the granulation tissue [125]. The accumulated adiposity might simply be a collateral bystander, and perhaps even the result of some regulatory mechanisms associated with the local control and remission of inflammation. For instance, the activation of PPARγ, the master regulator of adipogenesis [137], has also been described as downregulating IL-17-, TNFα-, and IL-6-mediated inflammation [138,139]. In addition, in certain microenvironments, both the adipogenic and osteogenic capacities of MSCs are simultaneously enhanced [140], and recent experimental data seem to suggest that BMP signaling through the BMP receptor 1A might contribute to explaining the abnormal adipogenic differentiation in the setting of AS [140].

Thus, remission of inflammation itself, whether spontaneous or treatment-induced, might be associated with PPARγ activation in immune and stromal cells within the hematopoietic bone marrow and may potentially also lead to local lipid accumulation and adipogenesis as a collateral result.

## 8. Systemic Bone Loss (Osteoporosis) in Ax-SpA

In addition to bone damage occurring at entheseal sites and joints, osteoporosis and low bone mineral density (BMD) may coincide, thus representing a major comorbidity [141]. Inflammation at the spine may also lead to localized bone loss and, thus, an increased risk of fracture, particularly at sites affected by BME [142,143].

In contrast to other inflammatory conditions such as RA, glucocorticoids are less frequently employed in SpA. Additionally, the profile of the patient with osteoporosis seems to differ, given that bone loss has been observed, for example, in young males [144,145].

Generalized bone loss in SpA has been correlated with immobilization as well as with its severity, duration of disease, and radiographic scores of ankylosis [146]. However, this possibility is not entirely adequate, given that bone loss has been observed to occur early in the course of disease [147,148]. Indeed, spinal inflammation can lead to trabecular bone loss and augmented risk of fracture: the presence MRI BME has been reported to increase the risk for low BMD by fivefold at both the spine and hip [143]. In addition, the close connection between BME and low BMD has been acknowledged even in non-radiographic ax-SpA [149].

In 2018, a meta-analysis reported a range for total osteoporosis, lumbar spine, and femoral neck osteoporosis of 11.7 to 34.4%, 11.6 to 19.1%, and 6.4 to 16.8%, respectively, as measured by DXA [150]. Moreover, recent data from the MEASURE1 phase III trial on secukinumab confirmed that a large proportion of patients with AS have reduced BMD [98]. Furthermore, it should be kept in mind that BMD may be overestimated. This is especially the case at the lumbar spine, where anteroposterior DXA may overestimate the BMD due to pathologic osteoproliferation and positively correlate with mSASSS (even if the same correlation was not significant with latero-lateral BMD, since the scan excludes vertebral edges, for the most part) [151].

At present, several risk factors for vertebral fracture (VF) have been recognized in patients with ax-SpA, including low BMD in the central and peripheral skeleton, older age, male sex, long duration of disease, impaired back mobility, augmented occiput-to-wall distance syndesmophyte formation, increased disease activity, and involvement of peripheral joints [152]. Regrettably, studies on the prevalence of VF in patients with SpA are not homogeneous [144], and there are substantial differences in populations studied and diagnosis [153,154,155]. The prevalence of VF in patients with SpA varies from 0% to 4% to up to 30 to 40% [155,156], with a higher risk (from threefold to sevenfold) compared to the general population [145,157]. To complicate the situation, the definition of VF also varies greatly across studies [141,155]. In the meta-analysis by Ramirez, the prevalence of fracture ranged from 11% to 24.6%, and there was substantial heterogeneity for all outcomes [150].

It is known that systemic inflammation is strongly associated with low BMD [148,158]. Its role in generalized bone loss in SpA is also supported by the demonstration of low cortical BMD, area, and thickness measured by high-resolution peripheral quantitative computed tomography [159]. This event has been reported to occur early in the course of disease. In addition, in a longitudinal study, Kim et al. reported that low BMD can independently predict structural progression [160]. Thus, this confirms the widespread and entangled implications of inflammation in SpA and bone health.

Genetic factors may also play a role. In this regard, transgenic (TG) HLA B27 rats have a lower mineral/matrix ratio as well as higher levels of proteoglycan and advanced glycation end products vs. wild-type rats [161], which are all characteristics related to impaired bone material properties.

An investigation in a similar rat model (TG HLA B27) also showed greater loss of bone density and strength with older age [162]. This appeared to be associated with greater bone remodeling and bone resorption, as demonstrated by the large number of osteoclastic precursors in bone marrow and the robust osteoclastogenic response to RANKL or TNFα [162]. In patients with SpA, dysregulation of the RANKL-RANK pathway has been reported, including increased concentrations of RANKL in serum (and higher levels of expression of intracellular RANKL in CD4+ and CD8+ T cells) [163]. In addition, autoantibodies to OPG were associated with low hip BMD and fractures in patients with SpA [164]. Lastly, dysregulation of Dkk-1, an inhibitor of the Wnt pathway, has been noted [44,45] in SpA/PsA. It has previously been observed that PTH is a possible cause of Dkk-1 levels in serum [43,45].

Taken together, these data appear to strongly advocate that the inflammation in SpA may be damaging to bone health by causing generalized bone loss due to systemic negative effects (similar to psoriasis and inflammatory bowel disease [165]) and by locally affecting sites of BME.

## 9. Systemic Bone Loss (Osteoporosis) in PsA

When considering the studies on PsA patients, the data are also heterogenous. Indeed, there is great diversity in the reported proportion of osteoporosis in PsA, ranging from 1.4% to 68.8% [166,167,168,169]. In a study from Frediani et al. published in 2001, a 30% prevalence of osteoporosis in PsA patients was reported; however, this was before the biological therapy era [168].

On the other hand, in a cross-sectional study published in 2018 on a cohort of Norwegian outpatients affected by PsA [170], osteoporosis as measured by DXA (defined as T-score ≤ −2.5) was only found in 6.4% of the patients. In the overall sample, gender-adjusted BMD (Z score) compared with the normative reference population data. To explain, at least in part, the wide range of the prevalence data reported in the different studies, the authors suggested a role for the biologic treatment adopted since early 2000. Disease activity of patients with PsA might be lower today than it was in the past decades, with greater focus on early diagnosis and more effective treatment options, and this might limit the burden of the disease on bone health. A meta-analysis published by Chandran et al. in 2016 confirmed the large degree of inconsistency among the various studies [171]: PsA patients had low BMD in almost half of the considered studies, while the other half negated the association [171]. When dealing with VF, in that same analysis, only four studies with data on occurrence of osteoporotic fractures in PsA were found [171]. Prevalence of fragility fractures ranged between 12% and 40% [171]. Small sample sizes were once again a limiting factor affecting the available studies. A more recent study, published in 2017, documented an elevated risk for incident fracture: OR 95% CI 1.16, 1.06; 1.27 [172]. In the same study, patients with mild psoriasis were reported to have similar risk for VF, or even higher when patients with severe psoriasis were considered (mild psoriasis, 1.07, 1.05; 1.10, and severe psoriasis, HR 95% CI 1.26, 1.15; 1.39) [172]. Finally, in two recent meta-analyses, Chen et al. showed that, while the DXA-assessed BMD seems not to be significantly reduced in PsA patients compared to controls [173], when a more sophisticated imaging technique such as HR-pQCT is adopted, PsA show a lower average volumetric BMD (total, trabecular, and cortical) [174]. Furthermore, the increased OR for fracture was confirmed: OR 95% CI 1.09, 1.06; 1.12 [173].

Studies focusing on the pathogenesis of generalized bone loss in PsA are lacking, and much of what was already discussed for ax-SpA may be valid too. However, some interesting speculation can be made on the cytokines involved in the development of the disease.

In particular, the role of IL-17 in the pathogenesis of psoriasis and PsA (and SpA as well) is well known and the subject of great scientific interest. Increased serum levels of IL-17 as well as increased numbers of Th17 cells have been documented in patients with active PsA [79]. As already broadly discussed, Th17 seems to have complex interactions with bone metabolism. Interestingly, in murine models, IL-17 blockade has been shown to be as effective as denosumab in preventing osteoporosis in ovariectomized rats [175]. However, data on the effects of biologic drugs (TNFi or secukinumab) on BMD in PsA patients, though promising, are very scarce and limited to hand BMD [176,177]

As we have seen, our knowledge of osteoporosis in ax-SpA and PsA is limited, but scientific interest in the topic is growing. We need further research to clarify the epidemiology of this comorbidity in the psoriatic disease as well as pathogenetic aspects and the implications for treatment. Furthermore, it is becoming clear that ax-SpA and PsA represent an independent risk factor for bone fragility. Fracture-risk assessment tools, such as FRAX, factor in the presence of RA in their calculation [178] but completely ignore both ax-SpA and PsA. On the other hand, in Italy, the open-source FRAX-derived fracture assessment tool (DeFRA, available at https://defra-osteoporosi.it/(accessed on 15 September 2023) includes SpA in its algorithm [179]. Recent data on osteoporosis treatment in patients with SpA suggest a suboptimal treatment coverage [180]; for all these reasons, we think that the widespread inclusion of SpA within these assessment tools would be helpful and necessary to grant our patients an adequate diagnosis and subsequent care for bone fragility.

## 10. The Effects of Pharmacological Treatment on Focal Bone Damage

### 10.1. NSAIDs

NSAIDs/COXIBs are usually recommended as a first-line drug class in the treatment of SpA [66,67]. In addition to its proinflammatory action, we already discussed the osteogenic potential of PGE2. Therefore, a possible effect on structural progression has been hypothesized. The data of NSAIDs on the reduction in BME score are very scarce. Two longitudinal studies documented an associated reduction in the Spondyloarthritis Research Consortium of Canada (SPARCC) score [181,182], though the absence of a control group and the limited magnitude of the improvement make it difficult to draw solid conclusions. One further study showed a significant reduction of MRI inflammation with naproxen therapy; however, this study also documented an accelerated development of fatty lesions [183]. Similarly, the data on structural progression show inconsistent results, and a systematic review published in 2019 concluded that NSAIDs are unlikely to significantly affect radiographic progression in AS patients [184].

### 10.2. TNFi

Biologics are currently the cornerstone pharmacological agents in the treatment of ax-SpA and have consistently been demonstrated to reduce clinical disease activity scores in these conditions [66,67]. The main monoclonal antibodies recommended belong to two different classes: TNFi and anti-IL-17 [66,67].

Unlike NSAIDs, the effectiveness of TNFi in reducing axial BME MRI has been investigated several times. However, in a systematic review with meta-analysis published in 2021, the authors concluded that there is still insufficient evidence to prove the benefit of TNFi on MRI inflammatory lesions of the spine and SIJ [185]. When different BME scores of the spine were pooled altogether, the analysis yielded a standardized mean difference of −72 95% CI −1.49; 0.04, *p* = 0.06, and at the SIJ, the subanalysis including the SPARCC SIJ score changes of the active groups compared to the control groups after 12 weeks yielded a significant mean difference of −3.19 95% CI −4.8; −1.58, *p* < 0.001 [185]. Albeit insufficient to prove an actual benefit, the sensitivity of such analysis may be hindered by significant limitations (i.e., the heterogeneity of the study populations, the different MRI scores adopted, the under-powered sample size to address imaging outcomes, and the natural flaring–remitting course of the disease).

Scrutinizing the effects of TNFi on structural progression is even trickier. The data from the first trials, which also considered TNFi and radiographic progression in AS (as secondary/exploratory endpoints), apparently failed to document a significant beneficial effect [186]. However, at that time, due to the limitations in the designs of the studies, a null finding was expected [186].

Meanwhile, the observation discussed previously—that syndesmophytes are more likely to develop after resolution of inflammation [187] and in the areas of fatty degeneration [188], and that successful treatment-induced resolution of inflammation was apparently associated with the development of fatty lesions [118,183,189]—led to an interesting debate and the formulation of the so-called “TNF-brake hypothesis” [190,191,192]. Based on the relationship between TNFα and the Wnt pathway that we extensively discussed in the previous paragraphs, the TNFα inhibition could, therefore, potentially be associated with a further decrease in Dkk-1 expression (albeit, this might not always be the case) and subsequently contribute to the worsening of bone osteoproductive damage.

Luckily, over the years, evidence accumulated, and, in 2019, a new systematic review with meta-analysis concluded that a possible effect of TNFi on radiographic progression could be present [193]. Interestingly, the same analysis, similarly to a less recent one [194], suggested that the benefit might become apparent only after several years of treatment (beyond two years) and especially when initiated in a timely manner (less than five years from the symptoms’ onset).

In fact, their results are in line with the pathobiological model that we proposed and with the imaging and [18F]fluoride studies presented, as already eloquently discussed by Maksymowych almost 10 years ago [191]. Confirmed by the [^18^F]fluoride PET/CT studies, there is evidence of significant osteoblastic activity already within the MRI BME lesions (Table S2). If we consider the effects of IL-17 (and IL-22?) on the early differentiation of MSCs towards the osteoblastic lineage, it is reasonable to hypothesize that within the inflammatory lesions, the microenvironment might already be primed for future granulation tissue development and bony proliferation. The resolution of inflammation, whether spontaneous or induced by TNFi, might therefore “remove the brake” on a process that has already been triggered and destined to happen; indeed, we already mentioned that the more advanced the inflammatory lesion is (i.e., CIL B), the more likely the development of syndesmophytes seems to be [117].

In a nutshell, for TNFi to exert their protective effect, it might be necessary a very early suppression of the inflammatory process, before excessive damage is achieved, and maladaptive osteogenic activity primed. Clearly, already advanced inflammatory lesions might be still doomed, and longer treatment periods might be needed for TNFis to show their benefit (in terms of prevention of inflammation and of its following consequences). In the same manner, the earlier the TNFi treatment is administered, the more likely that it might prevent the advancement of the local lesion to an irretrievable state.

In line with these speculations, in a very recently published study showing the four years post hoc MRI results of the phase III RCT on certolizumab pegol in ax-SpA, the authors confirmed that early and sustained suppression of inflammation was able to mitigate the risk of long-term fatty lesion development at the spine [195].

### 10.3. Anti IL-17

Presently, MRI data on IL-17 inhibitors have been published for secukinumab [196], ixekizumab [197], and bimekizumab in a very recent abstract presented at the 2022 EULAR congress. All three anti-IL-17 agents documented in their respective RCTs, in addition to their clinical effectiveness, demonstrated a significant reduction in BME MRI lesions. This is not surprising given the strong proinflammatory effects of IL-17.

In addition, data on the development of fatty lesions have been published only for secukinumab so far, showing a promising low conversion rate of BME lesions to fatty ones and the disappearance of about 30% of the fatty lesions observed before secukinumab treatment [198]. These findings are very preliminary, and their significance is still unclear; in our opinion, the findings deserve further attention and larger studies. Any effect on the regression of fatty lesions would be unprecedented in the treatment of SpA and potentially associated with relevant therapeutic implications such as the possibility of changing the natural history of such critically advanced pathologic lesions.

Concerning radiographic progression, secukinumab was the first biologic drug to show, in the setting of AS, a low overall rate of progression at two years, with 97.1% of patients in the overall population with no baseline syndesmophytes who remain syndesmophytes-free [199]. The data of the low rate of structural progression were also confirmed by a four-year extension study [200]. Similarly, a low two-year progression rate was also confirmed with ixekizumab, as very recently published [201].

Is there any data to suspect an “IL-17 brake” too? Currently, the data on Dkk-1 changes after treatment with anti-IL-17 are scarce. Unlike what has been observed with TNFi, in a longitudinal uncontrolled study, we observed an increase in serum Dkk-1 and sclerostin concentrations after treatment with secukinumab in a cohort of peripheral PsA patients [97]. Since the core of the “TNF-brake hypothesis” is the concealing of Wnt (and perhaps other pathways’?) inhibition, our finding seemed to point in an opposite direction, with a rise of Dkk-1 towards the serum concentrations of healthy controls. Further studies are necessary to confirm and corroborate these data, both in peripheral and axial disease.

Somewhat in line with what we described above, in an observational uncontrolled study on 20 PsA patients receiving treatment with secukinumab for six months, in which ultrasonography, MRI, and high-resolution peripheral quantitative CT (HR-pQCT) were performed, Kampylafka et al. observed that IL-17 inhibition was associated with the absence of any sign of both catabolic and anabolic structural progression [202]. Conversely, in a similar study on 41 PsA patients receiving treatment while either TNFi (N = 28) or methotrexate (N = 13), the development of erosions was arrested, but enthesophytes showed progression in both subgroups [203].

Clearly, comparisons of structural progression rates between recent RCTs and historical cohorts, or even with older RCTs, are heavily limited by several confounders [204]. Comparative data will hopefully be available shortly from the SURPASS trial (NCT03259074, completed on 29 November 2021). SURPASS is a randomized active-controlled head-to-head trial that compares the prevention of structural damage with secukinumab vs. adalimumab in biologic–naive AS patients [205] (31983056). The preliminary data of the SURPASS trial have been presented at the 2022 ACR (abstract number: L15) and seemingly failed to prove a significant difference in terms of radiographic progression between arms treated with secukinumab versus adalimumab biosimilar.

### 10.4. JAK Inhibitors

JAK inhibitors (JAKis) are an emerging class of drugs for the treatment of inflammatory arthritides and also represent a novel therapeutic intervention in the setting of SpA [206]. Blockade of the JAK/STAT and tyrosin kinase-2 (TYK2) cascade by different JAKis is associated with the inhibition of the signaling transduction of a broad range of cytokines involved in the pathogenesis of SpA, depending on the selectivity of each molecule [206].

Nevertheless, neither TNFα nor IL-17 directly signal via JAK pathways [207]. On the other hand, IL-23 signals via a JAK/TYk2 combination, IL-6 uses JAK1/JAK2, and IL-22 uses JAK1/TYK2 [206]. Therefore, the rationale for their use in SpA is to broadly inhibit inflammation and the downstream signaling implied in the IL-23- and IL-6-dependent activation of Th17 [207,208].

So far, only three JAKis have been investigated and licensed for the treatment of ax-SpA: tofacitinib, upadacitinib, and filgotinib [206]. In terms of MRI response, tofactitinib documented greater overall reductions in the SPARCC BME spine and SIJ scores than placebo [209]. Similar results were observed in the RCTs investigating upadacitininb [210] and filgotinib [211]. While these data seem promising, data on fatty lesions and structural progressions are currently lacking.

## 11. Effects of Pharmacological Treatment on Systemic Bone Loss in Patients with SpA

To date, no double-blind randomized clinical trial with antiresorptive or osteoanabolic agents has been conducted specifically in a SpA population; therefore, recommendations for the treatment and management of postmenopausal and male osteoporosis are applied in the clinical practice.

In 2014, our group investigated the effects of neridronate (an intravenous amino-bisphosphonate) in terms of disease activity in a six-month open-label parallel group study vs. infliximab in patients affected by AS [212]. Neridronate was successful not only in reducing disease activity, but also proved to be beneficial on the lumbar spine BMD [212].

Another study on pamidronate vs. golimumab showed similar improvements in BASDAI in the two groups, but with improvement in the BASFI and on MRI data on axial inflammation (SPARCC) limited to the golimumab group [213].

Data on NSAIDs are also limited. In a primary care-based nested case-control study, the risk of any clinical fracture was decreased in patients with SpA taking NSAIDs [157]. In addition, a large population-based public health database seemed to support a protective role of NSAIDs on the risk of clinical fractures in patients with SpA, with an observed increase only in those not on regular NSAIDs [214].

When considering TNFi, several meta-analyses reported benefits in terms of BMD on patients with AS [215,216,217,218]. Similarly, the effect of IL-17 blockade with secukinumab (data from the MEASURE1 RCT) showed a very promising BMD increase of 2.6% for lumbar spine, 0.9% for total hip, and 0.8% for femoral neck at one year, with corresponding percent changes at two years of 4.7% for lumbar spine, 0.5% for total hip, and 0.2% for femoral neck [98].

Unfortunately, specific studies scrutinizing the effects of these agents on vertebral fragility fractures are still lacking. A study retrospective published in 2013 on administrative databases analyzed a cohort of patients affected by psoriasis, SpA, or PsA [219]. In this cohort, there were no differences in the risk of combined fractures between new TNFi users and new users of a nonbiologic comparator.

**Figure 4:**
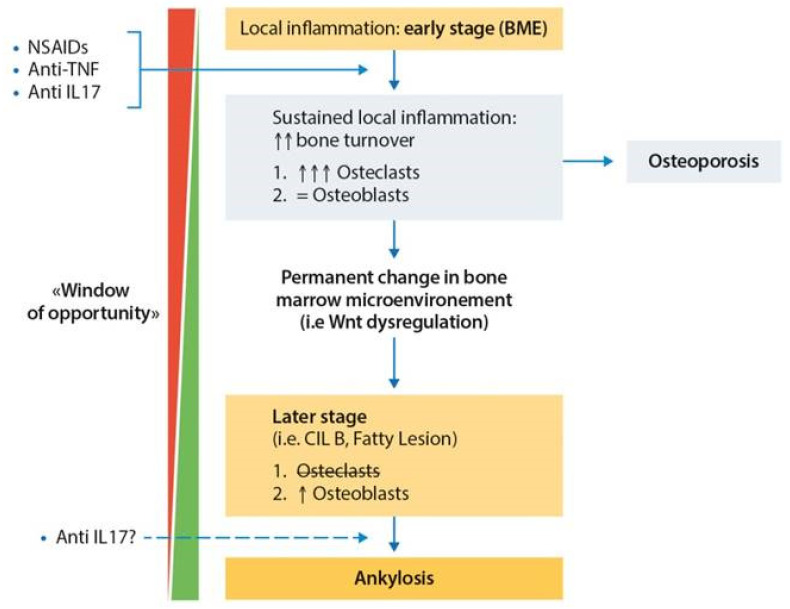
Time flow of the pathologic changes in spondyloarthritis. The first phase, mainly inflammatory and pro-osteoclast, is associated with lytic changes (i.e., vertebral corners and peripheral joint erosions). Systemic inflammation is also associated with systemic bone loss (osteoporosis). Involved cytokines and IL17 dysregulation may trigger and foster local microenvironment dysregulation, especially at entheseal sites, which may eventually lead to the development of syndesmophytes/enthesophytes. While the anti-inflammatory effects of several currently available treatments are well documented, a direct effect of anti-IL17 treatment on bone pathologic proliferation, while intriguing, has still to be clinically demonstrated (dashed line).

In conclusion, bone loss is a relevant comorbidity in SpA patients and must not be overlooked. Future studies on the possible benefits of osteoactive and anti-inflammatory agents (synthetic and biologic) in SpA patients need to focus on clinical and fracture outcome in order to improve the treatment and management of this condition. Figure 4 summarizes the time flow of the pathological focal and systemic changes in SpA and the possible benefits from the different drugs currently available.

## 12. Survey of Bone Metabolic Assessment in SpA from Italian bone Specialist Rheumatology Centers

### 12.1. Aim of the Survey

Given the importance of the link between SpA and systemic and local bone loss, we conducted a survey among several Italian rheumatology centers with the aim to explore the prescription habits in terms of imaging, DXA, and bone-related laboratory tests of patients affected with SpA receiving treatment with anti-IL-17 (secukinumab).

### 12.2. Methods

The survey was conducted among 11 Italian rheumatology units with high expertise in bone diseases. The survey was provided to each center and focused on the subgroups of SpA patients receiving treatment with secukinumab, a treatment registered limitedly for patients with SpA (PsA r-axSpA and nr-axSpA). Each center provided the aggregated data on the number of SpA patients treated (naïve or non-naïve to biologics), menopausal status, radiographic or non-radiographic disease, peripheral and/or axial involvement, and vitamin D supplementation. Data are reported as an absolute number or percentages, when appropriate. Differences in proportions were tested with a chi-squared test versus reference group (postmenopausal women), and the odds ratio (OR) was calculated with a relative 95% confidence interval (CI).

### 12.3. Results

Data on 1076 patients, 489 males and 587 females (of whom 298 were postmenopausal), were retrieved. Of the total, 514 were naïve and 562 non-naïve to biologics. All patients with axial involvement had an axial X-ray and/or an MRI scan. The type of involvement of the sample and subgroups are reported in Supplementary Table S2.

In the subjects affected by exclusive p-SpA, axial X-rays had been performed in 76% (122/161) of the postmenopausal subgroup, 63% (72/114) of the premenopausal subgroup (*p* < 0.05 vs. postmenopausal women), and 62% (94/151) of the male subgroup (*p* < 0.05 vs. postmenopausal women).

Supplementary Table S3 reports the prevalence in the prescription of the DXA scan of the total sample and shows divisions according to gender and premenopausal or postmenopausal status, Supplementary Table S4 reports the data on the prevalence of BTMs and 25(OH)D prescription, while Supplementary Table S5 reports the data on vitamin D supplementation. Concerning osteoactive treatment, only a small proportion of patients (4.3%) was on therapy. Of these 46 patients, 15 were males and 31 were women, of whom 25 were postmenopausal. The most common treatment was bisphosphonates (42/46). Out of the 42 patients, 28 were treated with oral and 14 with intravenous formulations. Only 4 of 46 patients were administered denosumab.

### 12.4. Discussion of Survey Data

In this survey, we retrieved interesting data regarding both the diagnostic procedures for the assessment of bone health and its therapeutic management. The prevalence of DXA examination was significantly lower in the premenopausal and in the male subgroups. It is plausible that the increased use of radiology in postmenopausal women was also driven by a greater interest in skeletal health due to gender and older age. Thus, about half of the postmenopausal women had at least one DXA evaluation at baseline, and more than one out of three at least a DXA scan during the follow-up.

Concerning biochemical assessment, the more commonly adopted bone marker was ALP (29% of the overall sample), though it is plausible that the use of ALP could also be driven by non-bone-related concerns. The use of a more specific BTMs (i.e., CTX or bone-ALP, PINP, etc.) was less common (16%).

Interestingly, the serum 25(OH)D concentrations resulted in the most frequent bone-related laboratory tests, although with a significantly higher adoption in the postmenopausal subgroup. Consequently, a large proportion of patients were treated with vitamin D supplements, especially women (both premenopausal and postmenopausal), but over half of the men as well. The number of patients treated with active agents was too small to be critically evaluated.

These data confirm, as expected, that most of the attention, in terms of the effect of bones on bone health, is paid to postmenopausal subjects. Given, however, the relationship between spondyloarthritis and impaired bone health, in our opinion, the screening procedures are still suboptimal, even in rheumatology centers with a specific interest in bone metabolic disease. Similar considerations could be made for biochemical assessment—except for vitamin D assessment, which seemed to be widespread—and, as a consequence, for its supplementation.

## 13. Conclusions

While the relationship between systemic inflammatory conditions and systemic and local bone damage has been extensively studied in RA, the same is not equally true for SpA. It is acknowledged that SpAs are associated with a significant burden of disease that is driven by pain and functional disability caused by active inflammation. In addition, with varying degrees of severity depending upon the intertwined relationships between genetic susceptibility, immune dysregulation, and environmental factors, bone damage may ensue.

The present review explored the biological mechanisms underlying the relationship between inflammatory dysregulation, structural progression, and osteoporosis in this diversified family of conditions. Firstly, we addressed the biology and metabolic regulation of bone, with a particular focus on SpA. Additionally, we discussed the significance of key regulatory factors in osteogenesis, including BMPs, the Wnt system, and Hh signaling. Furthermore, we analyzed the role of major cytokines involved in the pathogenesis and amplification of inflammation, such as IL-17, TNFα, IL-23, and IL-6, as well as their interaction with the bone microenvironment.

Drawing on these premises, we introduced a pathophysiological model aimed at summarizing the intricate interplay between immune system dysregulation, its consequential impact on bone metabolism, and the progression of damage, assessed through various available techniques such as X-rays, MRI, and [18F]PET/CT.

However, while the advancement of local bone damage, characterized by neoformative changes like syndesmophytes and enthesophytes as well as resorptive features like erosions, has always been considered a hallmark of SpA, the significance of systemic bone damage has frequently been underestimated. Despite this, a wealth of evidence now strongly demonstrates the accelerated bone loss and heightened risk of fragility fractures in SpA. This consolidates the primary available data in this context, encompassing both ax-SpA and PsA.

Subsequently, we scrutinized and provided commentary on the existing literature regarding the impact of various drugs employed in these conditions, including NSAIDs, TNFi, anti-IL-17 agents, and JAK inhibitors, with a focus on both localized and systemic damage. Lastly, we presented findings from a multicenter survey detailing prescription patterns regarding imaging, DXA, and bone-related laboratory tests for patients with SpA undergoing treatment with an anti-IL-17 agent (secukinumab). This survey engaged 11 highly specialized rheumatology centers that are proficient in managing SpA and osteometabolic disorders. The overarching outcome underscores the heterogeneity and selective nature of bone screening and assessment procedures for this unique population. Even within dedicated facilities, attention tends to be concentrated on a minority of this group, primarily postmenopausal women, as anticipated. In our opinion, it may be the time for a call to action to increase the interest and focus on the diagnosis and management of this significant, troublesome liaison.

## Figures and Tables

**Table 1:** Table summarizing the effects of the main mediators involved in the pathogenesis of bone damage in SpA. Abbreviations: BMPs, bone morphogenic proteins; Dkk-1, Dickkopf-related protein 1; OPG, osteoprotegerin; PGE2, prostaglandin E2; RANKL, receptor activator of nuclear factor-kappa beta; TNFa, tumor necrosis factor a.

Mediator	Role in the Development of Bone Damage in SpA	REF
IL-17	Proinflammatory cytokine, stimulates bone resorption. Concurrently, can positively regulate the early phase of osteoblastogenesis. In addition, acts upon the Wnt and BMPs pathways and may upregulate IL-22 expression.	[68,78,82,83,94,95,96,100]
TNFα	Proinflammatory cytokine, stimulates bone resorption acting synergically with RANKL. Regulates Dkk-1 and sclerostin expression.	[16,52,53,54,107,108]
IL-6	Proinflammatory cytokine. May promote Th17 differentiation.	[68]
IL-23	Proinflammatory cytokine, among the key drivers of Th17 differentiation.	[79]
IL-22	May enhance the osteogenic capacity of MSC within a proinflammatory environment.	[81]
RANKL	The key driver of osteoclast differentiation and activation.	[10]
OPG	Decoy receptor and RANKL antagonist.	[14]
Dkk-1	One of the main inhibitors of the Wnt canonical pathway. Also defined as the “master regulator of joint remodelling”.	[26,34]
Sclerostin	Similar to Dkk-1.	[26]
BMPs	Alongside with the Wnt pathway, a fundamental regulator of osteogenesis. Dysregulated in SpA.	[26,33]
PGE2	Proinflammatory mediator with marked vasodilating properties. Upregulates Th17 differentiation and IL-17 secretion; may upregulate BMP, Wnt, and Hh signalling.	[84,86,87,88]

## Supplementary Materials

The following supporting information can be downloaded at: https://www.mdpi.com/article/10.3390/ijms241914924/s1.
