# Supplementary material for: Osteoimmunology of Spondyloarthritis
Source: Int J Mol Sci. 2023 Oct 5;24(19):14924. doi: 10.3390/ijms241914924 (PMC10573470; doi:10.3390/ijms241914924)
Supplement: Supplementary file 1 [file ijms-24-14924-s001.zip › ijms-2566336-supplementary.pdf]

Supplementary Table S1. Main studies investigating [^{18}F]fluoride PET/CT.

Author	Study type	N	Follow-up	Results	Comments
Lee SJ, 2020	Longitudinal	28	4.5 y	The baseline LBR of posterior joints had significant (weak) positive correlation with follow-up BASDAI score (r=0.394, P= 0.042).	
Buchbender C, 2015	Cross-sectional	10		BME rather than chronic changes (FL) is associated with osteoblastic activity, while the combination of BME and FL showed the highest ^{18}F-f uptake.	
Kim K, 2020	Longitudinal	27	2 y	The baseline SUV max of the spine (but not of the SIJ) predicted BASDAI 50 response at 2 years to anti-TNF (OR with 95%CI: 14.00 1.37–14.29, p=0.009)	
Kim K, 2020	Cross-sectional	12		Significant correlation between the SUV_{mean} of the vertebral corners and DXA measured BMD at the lumbar spine (r=0.402 p = 0.005).	The correlation is likely to express the artefactual increase of BMD due to pathologic bone formation.
Seung SM, 2020	Retrospective	68 patients with LBP. 49 satisfied ASAS criteria, 19 did not (and were considered as controls)		More common PET-positive findings in the ASAS-classified group than in the control group (79% vs 15%, p<0.001).	
Fischer DR, 2012	Cross-sectional	10		Increased ^{18}F-f uptake in PET/CT is “only modestly” associated with bone marrow edema on MRI in the spine and SIJ (Cohen’s K value = 0.25, p value not provided)	
Idolazzi L, 2016	Cross-sectional	29		The number of ^{18}F-f PET/CT positive sites was significantly higher in patients with severe functional impairment and higher disease activity and was positively related to both BASDAI (r = 0.336; P = 0.036) and ASDAS (r = 0.408; P = 0.014)	
Strobel K, 2020	Cross-sectional	15 AS, 13 LBP controls		The AUC for sacroiliitis at the ^{18}F-f PET/CT was 0.84.	Criteria for the definition of “sacroiliitis” at PET/CT not provided
Raynal M, 2019	Cross-sectional	23		No correlation between ^{18}F-f PET/TC findings and CT findings (erosion, ankylosis scores). Significant correlation between the 18 F-f PET activity score and the inflammation score SPARCC (ICC [95% CI] = 0.61 [0.26; 0.82]; p = 0.001.	FL not assessed
Ouichka R, 2019	Cross-sectional	23		Abnormal uptake by the SIJ on ^{18}F-f PET is more frequent (87.0%) than inflammatory	

			(43.5%) and structural sacroiliitis (65.2%) on MRI. The ^{18}F-f PET activity score and SUVmax had good correlations with inflammatory sacroiliitis but not with structural lesions on MRI.	
Toussirot E, 2015	Cross-sectional	15	At SIJ, good concordance between active inflammatory areas depicted on ^{18}F-f PET-CT and MRI inflammatory lesions. For spinal lesions, the number of fluoride lesions on PET-CT scans largely exceeded those detected by spinal MRI (33 vs. 4). There was also a relationship between SIJ MRI score and SIJ/S SUV ratio.	Letter to the editor, full data not shown
Lee SG, 2015	Cross-sectional	12	A type A CIL (OR=3.2, 95% CI=1.6-6.5, p=0.001), type B CIL (OR=59.9, 95% CI=23.5-151.5, p<0.001) and syndesmpophyte (OR=21.8, 95% CI=5.5-85.2, p<0.001) were significantly associated with an increased ^{18}F-f PET/CT uptake lesion.	

Bone marrow edema; CIL, corner inflammatory lesion; DXA, dual-energy X-ray absorptiometry; FL, fatty lesions; LBR, lesion-to-background ratio; SIJ, sacroiliac joints.

Supplementary Table S2. Clinical and radiographic features of the explored sample. AS, ankylosing spondylitis; nr-axSpA, non-radiographic axial spondyloarthritis; p-SpA; peripheral spondyloarthritis.

	AS	Nr-axSpA	SpA with both axial and peripheral involvement	Exclusively p-SpA involvement	Total
**Males**	148	66	124	151	489
**Pre-menopausal women**	59	43	73	114	289
**Post-menopausal women**	32	17	88	161	298
**Total**	239	126	285	426	1076

Supplementary Table S3. Prevalence of DXA assessment at baseline and at follow-up. DXA; dual-energy X-rays absorptiometry; OR, odds ratio, CI, confidence interval.

	DXA at the beginning of therapy	OR (95% CI) vs postmenopausal women	DXA at follow-up	OR (95% CI) vs postmenopausal women
**Males**	20%	0.28 (0.21-0.39) [a,b]	14%	0.55 (0.39-0.80) [a,b]
**Women, pre-menopausal**	30%	0.48 (0.34-0.67) [a]	21%	0.72 (0.46-1.06)
**Women, post-menopausal**	47%	Reference	38%	Reference

[a] p<0.0005 vs. reference; [b] p<0.005 vs. pre-menopausal women.

Supplementary Table S4. Prevalence of ALP, other BTMs and 25(OH)D assessment at baseline and at follow-up. ALP, alkaline phosphatase; BTM, bone turnover markers; 25(OH)D, serum 25 hydroxy vitamin D concentration; OR, odds ratio, CI, confidence interval.

	**ALP**	**OR (95% CI) vs postmenopausal women**	**Other BTMs**	**OR (95% CI) vs postmenopausal women**	**25(OH)D assay**	**OR (95% CI) vs postmenopausal women**
**Males**	19%	0.36 (0.26-0.49) [a,b]	7%	0.19 (0.12-0.29) [a,b]	40%	0.43 (0.32-0.57) [a,b]
**Women, pre-menopausal**	35%	0.80 (0.58-1.13)	20%	0,72 (0.46- 0.99) [c]	51%	0.68 (0.49-0.94) [c]
**Women, post-menopausal**	40%	Reference	27%	Reference	61%	Reference

[a] p<0.0005 vs reference; [b] p<0.005 vs. pre-menopausal women; [c] p<0.05 vs reference.

Supplementary Table S5. Prevalence of vitamin D supplementation. OR, odds ratio, CI, confidence interval.

	**Supplemented with vitamin D**	**OR (95% CI) vs postmenopausal women**
**Males**	60%	0.23 (0.15-0.33) [a,b]
**Women, pre-menopausal**	86%	0.94 (0.58-1.51)
**Women, post-menopausal**	87%	Reference

[a] p<0.0005 vs. reference; [b] p<0.005 vs. pre-menopausal women.
